# Demonstrating the Qualitative Differences between Semantic Aphasia and Semantic Dementia: A Novel Exploration of Nonverbal Semantic Processing

**DOI:** 10.3233/BEN-2012-110200

**Published:** 2013

**Authors:** Krist A. Noonan, Peter Garrard, Elizabeth Jefferies, Sheeba Eshan, Matthew A. Lambon Ralph

**Affiliations:** ^1^School of Psychological SciencesUniversity of ManchesterManchesterUK; ^2^Department of PsychologyUniversity of YorkYorkUK; ^3^Stroke and Dementia Research CentreSt George’s University of LondonLondonUK

**Keywords:** Semantic representation, semantic control, aphasia, semantic dementia, feature reality task

## Abstract

Semantic dementia (SD) implicates the anterior temporal lobes (ATL) as a critical substrate for semantic memory. Multi-modal semantic impairment can also be a feature of post-stroke aphasia (referred to here as “semantic aphasia” or SA) where patients show impaired regulatory control accompanied by lesions to the frontal and/or temporo-parietal cortices, and thus the two patient groups demonstrate qualitatively different patterns of semantic impairment [1]. Previous comparisons of these two patient groups have tended to focus on verbal receptive tasks. Accordingly, this study investigated nonverbal receptive abilities via a comparison of reality decision judgements in SD and SA. Pictures of objects were presented alongside non-real distracters whose features were altered to make them more/less plausible for the semantic category. The results highlighted a number of critical differences between the two groups. Compared to SD patients, SA patients: (1) were relatively unimpaired on the two alternative forced choice (2AFC) decisions despite showing a comparable degree of semantic impairment on other assessments; (2) showed minimal effects of the plausibility manipulation; (3) were strongly influenced by variations in the regulatory requirements of tasks; and (4) exhibited a reversed effect of familiarity–i.e., better performance on less commonly encountered items. These results support a distinction between semantic impairments which arise from impaired regulatory processes (e.g., SA) versus those where degraded semantic knowledge is the causal factor (e.g., SD). SA patients performed relatively well because the task structure reduced the requirement for internally generated control. In contrast, SD patients performed poorly because their degraded knowledge did not allow the fine-grained distinctions required to complete the task.

## 1. Introduction

Semantic cognition is fundamental to our everyday lives because it allows us to activate and utilise conceptual knowledge in a flexible, time- and context-appropriate fashion. This requires comprehension of objects in terms of their core similarities and differences and not just their superficial characteristics [2, 3]. In addition, semantic cognition (rather than semantic memory alone) requires executive regulation to focus on which specific aspects of our rich semantic database are appropriate for the task in hand [4, 5]. Semantic dementia (SD), a condition associated with bilateral anterior temporal lobe (ATL) degeneration, is the purest example of degraded semantic knowledge [6]. SD patients exhibit a frequency graded loss of semantic knowledge across all testing modalities

ISSN 0953-4180/13/$27.50 © 2013 – IOS Press and the authors. All rights reserved

(e.g., pictures, words, sounds, smells) and gradually lose the fine-grained knowledge required to distinguish between similar concepts [7–9]. This supports the idea that the ATL bilaterally is a critical substrate for semantic representations and SD represents a 'dimming' of semantic acuity [10]. Multimodal semantic impairments can also emerge in the context of stroke aphasia (referred to here as semantic aphasia or SA) where damage is restricted to the left hemisphere, in areas outside the ATL [1]. Recent investigations show that these patients do not have degraded knowledge, but that their semantic performance is modulated by the requirement for regulatory control [5,11–13]. These earlier studies utilised verbally-based tasks in order to manipulate key control-related factors or used more inherently complex open-ended assessments of object use. Given that both patient groups exhibit nonverbal as well as verbal comprehension impairment, a dedicated comparison of the nature of nonverbal receptive skills in SD and SA is required. Accordingly, this paper directly compares both patient groups on a multimodal feature reality task to examine the hypothesis that, in line with the results from verbal assessment, the two patient groups present with qualitatively different patterns of nonverbal semantic performance.

Since the earliest observations of SD it has been recognised that conceptual loss is graded in nature [14]. Aside from the benefits derived by frequently encountered items, concepts with the highest proportion of shared features (i.e., the most *typical* concepts from a semantic category) tend to survive the longest and exert a strong influence on residual semantic processing. In picture naming, this is evident in patients' errors, where the names of frequent and typical category members are often erroneously produced and inappropriately over-extended (e.g., *cat* for a picture of a *squirrel*). SD patients also produce many superordinate errors, where they identify pictures as belonging to a particular category but are unable to produce the specific name (e.g., *squirrel* → "*animal*"). Recent computational models of the ATL provide a principled explanation for these findings. Rogers et al. [15] conceptualised the bilateral ATL as a semantic hub that extracts amodal structure from multimodal experience (see also [3,16]). Importantly, in this model, concepts with many shared features contribute the most towards change in connection weights and lead to the entrenchment of broad category boundaries which are highly resistant to damage [17]. Distinctive features (e.g., a *squirrel's* bushy tail; an *elephant's* trunk) that allow discrimination between similar category members are the most vulnerable to damage because they lack the redundancy provided by consistent collateral activation. Many of the key typicality findings in the SD literature have been simulated by this model including patterns of longitudinal naming performance [i.e., the way that responses become more general over time: 8]) and the tendency to omit specific features in delayed picture copying [e.g., drawing a camel without its hump; 15].

In line with this framework, Garrard and Carroll [18] demonstrated that SD patients were poor at making alternative forced-choice reality decision judgements when the distracter was characterised by a feature that was not possessed by the pictured item, but was applicable to the item's semantic neighbours. More specifically, SD patients had difficulty choosing the real object when the non-real distracter items had a colour, sound or environmental context consistent with other real semantic items (e.g., colour condition: greater difficulty choosing a *red strawberry* when accompanied with a *green strawberry*, than when paired with a *blue strawberry*). Critically, impairment on this task correlated strongly with background tests of semantic knowledge and patients were highly consistent across conditions, supporting the idea that the progressive 'dimming' of amodal semantic representations was the causal factor in each of these tasks.

The nature of the semantic impairment in SA is quite different. Although patients with SA can show an equivalent degree of impairment across verbal and non-verbal versions of semantic tasks, degraded representations do not appear to be the causal factor. In contrast to SD patients, individuals with SA (1) show variable performance across tests requiring different types of semantic judgements [e.g., naming vs. associative decisions; 1]; (2) are sensitive to refractory/access variables [i.e., speed of presentation, item repetition and semantic blocking; 12]; (3) exhibit minimal or no effects of frequency/familiarity [12]; (4) demonstrate strong effects of cues in picture naming, object use and synonym judgement [1,5,11,13]; and (5) have lesions in prefrontal (PFC) and/or the pMTG-to-angular gyrus region (temporo-parietal junction (TPJ), sparing the ATL [1,19]. These results indicate that, following lesions to the PFC or TPJ, patients develop increased sensitivity to competition within the semantic system: although their semantic representations are not severely degraded (and in fact may not be impaired at all), access to this knowledge is deregulated following impaired semantic control. This pattern has been replicated by repetitive transcranial magnetic stimulation studies in control participants [20]. Functional neuroimag-

Table 1
Demographic information for SA patients

| Patient | Age | Sex | Education (leaving age) | Neuroimaging summary | PFC | TPJ | Aetiology of CVA | Aphasia type |
|---|---|---|---|---|---|---|---|---|
| JD | 81 | M | 16 | Infarction of putamen and internal capsule. Compression of L lateral ventricle. | † | † | Haemorrhage | Mixed transcortical |
| NY | 65 | M | 15 | L frontal-temporal-parietal | √ | √ | | Conduction |
| PG | 62 | M | 18 | L frontal and capsular (CT) | √ | † | Subarachnoid haemorrhage | TSA |
| HN | 78 | M | 14 | L occipital-temporal | × | √ | Ischemia | Anomic/TSA |
| SC | 78 | M | 16 | L occipital-temporal (& R frontal-parietal) | × | √ | Haemorrhage | Anomic/TSA |
| BB | 57 | F | 16 | L frontal & capsular (CT) | √ | √ | Subarachnoid haemorrhage | Mixed transcortical |
| ME | 39 | F | 16 | L occipital-temporal | × | √ | Subarachnoid haemorrhage | TSA |
| KA | 76 | M | 14 | L frontal-temporal-parietal | √ | √ | Thomboembolic/partial haemorrhage | Global |
| LS | 73 | M | 15 | L temporal-parietal-frontal | √ | √ | | TSA |

Patients are arranged in order of composite semantic score (see Table 2). Aphasia classifications were derived from the Boston Diagnostic Aphasia Examination [BDAE; 48]. JD withdrew from the study before the BDAE could be administered therefore classification was based on background assessments of fluency, repetition and comprehension. TSA = Transcortical sensory aphasia. Neuroimaging summaries are based on visual inspection of CT/MRI scans, except in the cases of PG and JD where only written reports of clinical scans were available. PFC = lesion involves left prefrontal cortex; TPJ = lesion involves left temporoparietal junction; †= neuroradiological information is not sufficient to make a definitive statement regarding the extent of cortical damage.

ing studies of healthy participants are also consistent with this view. For example, the process of orientating attention to particular semantic categories, processing the non-literal meaning of metaphors and retrieving the less dominant meanings of ambiguous words gives rise to activation in both TPJ and inferior PFC regions [21–23]. Anatomically, these two cortical areas (i.e., PFC and TPJ) are strongly connected by the arcuate and superior longitudinal fasciculi, and may form a distributed but functionally coherent system [24,25].

Although the nature of the semantic impairment in SD and SA is very different, reflecting degraded semantic representations and impaired semantic control respectively, both types of impairment affect nonverbal as well as verbal comprehension. Garrard and Carroll [18] used a series of novel, nonverbal reality decision judgements to probe the nature of nonverbal semantic processing in semantic dementia and in this study we compared these results to those obtained for a group of SA patients. In doing so, this study is the first to investigate the impact of *feature* typicality in SA. As noted above, SD patients lack knowledge of the specific features of objects and will base their decisions on more general information. For this reason, SD patients are highly susceptible to 'plausible' distracter items which have a strong overlap with the target and other semantic neighbours [18]. SA patients should be less vulnerable to 'plausible' distracters because they retain intact semantic representations that include the unique features of items. In addition, SA patients will benefit from the use of a 2AFC format because this form of assessment reduces the requirement for internally-generated control and minimises competition between potential response choices. These patients may therefore be relatively unimpaired when making reality judgements, even when they are matched to SD patients for other semantic test results.

## 2. Participants

A total of nine SA patients took part in the study. Their ages ranged from 39 to 81, with a mean of 67 years. On average the patients completed eleven years of education. All of the patients had chronic impairments resulting from a CVA at least one year prior to testing. Patients were selected to show multimodal semantic deficits affecting the comprehension of words and pictures (see Table 2). The group included patients with fluent and less fluent profiles (Table 1 provides background aphasialogical and demographic information). Neuroradiological investigations (where available) confirmed that the SA patients had left hemisphere lesions in the PFC and/or the TPJ regions. Figure 1 presents MRI scans for SC, NY, HN, ME and LS as well as CT scans for BB and KA. MRI was not performed in PG or JD due to contraindications, although

Table 2
Background neuropsychology for the Semantic Aphasic patients

| | Max | Normal cut off | JD | NY | PG | HN | SC | BB | ME | KA | LS |
|---|---|---|---|---|---|---|---|---|---|---|---|
| Semantic tests | | | | | | | | | | | |
| Composite semantic score | | | 0.97 | 0.91 | 0.84 | 0.65 | 0.55 | −0.20 | −1.07 | −1.20 | −1.45 |
| WPM | 64 | 62 | 64 | 60* | 58* | 50 | 59* | 54* | 50* | 26* | 37* |
| Picture Naming | 64 | 59 | 49 | 55* | 46* | 50 | 28* | 10* | 5* | 0* | 5* |
| CCTp | 64 | 51 | 38 | 36* | 44* | 44 | 46* | 38* | 13* | 46* | 16* |
| Background Neuropsychology | | | | | | | | | | | |
| VOSP Screening | 20 | 15 | 20 | 19 | 20 | 19 | 20 | 20 | 19 | 20 | 18 |
| VOSP Position Discrimination | 20 | 18 | 20 | 20 | 20 | 19 | 17* | 18 | 15* | 14 | 16* |
| VOSP Number Location | 10 | 7 | 10 | 10 | 9 | 9 | 0* | 8 | 2* | 6 | 8 |
| Corsi span forward | | | 4 | 4 | 5 | 4 | 3 | 5 | 3 | 5 | 3 |
| Corsi span backward | | | 3 | 4 | 2 | 4 | 3 | 0 | 2 | 2 | 2 |
| Digit span forward | – | 5 | 5 | 3* | 6 | 6 | 6 | 5 | 6 | 0* | 4* |
| Digit span backward | – | 2 | 2 | 2 | 2 | 2 | 2 | 0* | 3 | NT | 1* |
| Brixton Spatial Anticipation Test (correct) | 55 | 28 | 28 | 34 | 26* | 27* | 25* | 23* | 11* | 6* | 14* |
| TEA: elevator counting (no distraction) | 7 | 6 | 7 | 3* | 3* | 7 | 7 | 4* | 7 | TA | 3* |
| TEA: elevator counting (distraction) | 10 | 3 | 6 | 2* | 0* | 9 | 1* | 0* | 9 | NT | 2* |
| Hayling sentences: A (errors) | 15 | – | 1 | 3 | 3 | 1 | 5 | 3 | 1 | TA | 8 |
| Hayling sentences: B (errors) | 15 | – | 6 | 8 | 1 | 12 | 6 | 6 | 3 | TA | 6 |
| Raven's coloured matrices (correct) | – | – | 30 | 28 | 27 | 20 | 28 | 32 | 16 | 17 | 24 |
| WCST (no. categories) | 6 | 1 | 1 | 2 | 0* | NT | 6 | 1 | 0* | 1 | 0* |

Patients are arranged in order of their composite semantic scores derived from the three background tests of semantic processing (i.e., WPM, picture naming, CCTp). WPM = word to picture matching; CCTp = picture version of the Camel and Cactus Test [7]; VOSP = Visual Object and Space Perception battery [26]; TEA = Test of Everyday Attention [32]; WCST = Wisconsin Card Sorting Test [30,31]. *denotes impaired performance. NT = not tested. TA = testing abandoned.

written reports of previous CT scans were available for both cases.

The SA group was compared with 12 SD cases recruited from specialist memory clinics in London and Cambridge. Ages ranged from 56 to 73, with a mean of 64 years. On average, patients completed 13 years of education. These patients were first described by Garrard and Carroll [18]. Their data are re-reported here in order to license a direct comparison to those obtained for the SA patients. All SD cases fulfilled all of the published criteria for SD [6]. Patients presented with word-finding difficulties in the context of fluent speech and were impaired on tests of single word comprehension and semantic knowledge. In contrast, phonology, syntax, nonverbal reasoning and visuo-spatial processing were relatively well preserved. They all showed relatively circumscribed atrophy of the anterior temporal lobes.

Twelve control participants were selected from a volunteer database to match as closely as possible the age and educational level of the patient groups. This group ranged in age from 41 to 78, with a mean of 67 years. Controls completed, on average, 12 years of full-time education.

## 3. Background neuropsychological assessment

### 3.1. General neuropsychological tests

Visual-spatial processing in both groups was assessed using the Visual Object and Space Processing battery [VOSP: 26]. Working memory in the SA group was examined using forward and backward digit span [27] and the Corsi block-tapping task [28]. A range of additional tests were used to assess executive/attentional functions in the SA group: (1) the Ravens Coloured Progressive Matrices test of nonverbal reasoning [29], (2) the Wisconsin Card Sorting test [30,31], (3) Elevator Counting with and without distraction from the Test of Everyday Attention [32], (4) the Brixton Spatial Rule Attainment task [33] and

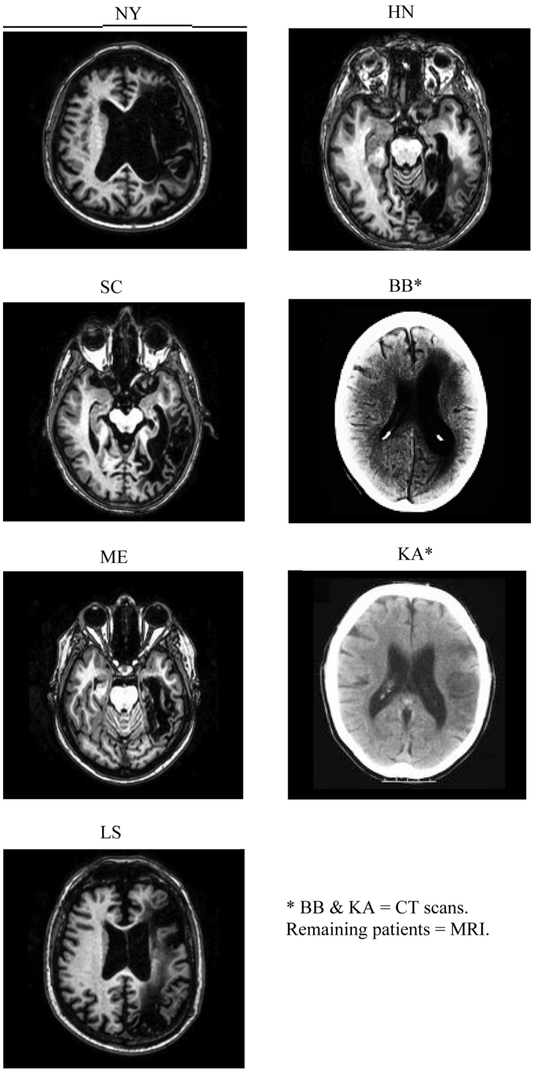

Fig. 1. Neuroimaging for the SA patients.

(5) the Hayling Sentence Completion test [33] which contrasts the production of appropriate sentence endings (e.g., "It is hard to admit when one is... *wrong*") with nonsensical endings that require the prepotent word to be suppressed (e.g., "Most sharks attack very close to... *cups*").

### *3.2. Results*

Tables 2 and 3 presents the background data for SA and SD patients, respectively. Visual-spatial processing was relatively preserved in the SA group: only three patients failed one or more subcomponents of the VOSP. Evidence of mild visual-spatial problems was also evident in three SD patients. Some SA patients showed additional problems on the memory span tasks (i.e., corsi block-tapping and digit span). However, all SA patients showed evidence of impaired executive/attentional processing.

### *3.3. Background semantic memory assessments*

The presence of multimodal semantic impairment was assessed using a battery of semantic tests which tapped different input and output modalities for the same 64 items [7]. There were four test components:

1. Spoken word-picture matching (WPM): The patients were required to match a verbally presented word to a target picture presented alongside nine semantically related foils. The pictures were black and white line drawings taken from the Snodgrass and Vanderwart [34] corpus.
2. Picture naming: The patients named these drawings presented individually.
3. Camel and Cactus Test [CCT'; Bozeat et al. 7]: This test of semantic associations is similar to the Pyramid and Palm Trees test [35]. Patients were asked to decide which of four pictures was most associated to a probe picture: e.g., "camel" with "cactus", "rose", "tree" or "sunflower"?

A composite score reflecting each patient's overall semantic abilities was derived from these three semantic tests using factor analysis. This score was computed separately for each patient group, with larger numbers representing better semantic performance. All tables are ordered by this score.

### *3.4. Background semantic test results*

Tables 2 and 3 show that every patient, regardless of group, was impaired on all background semantic tasks. SA and SD patients showed no quantitative difference in their level of impairment across picture naming, WPM and CCTp (all $t$ values $< 1$; see Fig. 2). As expected given their amodal semantic breakdown, SD patients showed strong correlations across all three semantic tasks ($r > 0.84$, $p < 0.001$). In line with underlying poor executive-semantic control [1], the SA patients were far less consistent, showing a correlation between the two tasks which required identity matching (e.g., WPM and naming: $r = 0.72$, $p < 0.03$) but not between CCTp and WPM or CCTp and naming ($r > 0.45$, $p > 0.2$). SA patients also showed correlations between their semantic test performance and measures

Table 3
Background neuropsychology for the Semantic Dementia patients

| | Max | Normal cut off | JM | NG | BG | AN | VH | RW | DW | TW | WM | IB | FO | AT |
|---|---|---|---|---|---|---|---|---|---|---|---|---|---|---|
| Semantic tests | | | | | | | | | | | | | | |
| Composite semantic score | | | 1.06 | 1.02 | 0.97 | 0.82 | 0.49 | 0.28 | 0.05 | −0.80 | −0.95 | −1.41 | −1.51 | – |
| WPM | 64 | 62 | 61* | 59* | 61* | 62 | 59* | 56* | 49* | 38* | 18* | 19* | 11* | NT |
| Picture Naming | 64 | 59 | 48* | 60* | 41* | 41* | 38* | 39* | 33* | 8* | 10* | 5* | 1* | 0* |
| CCTp | 64 | 51 | 54 | 46* | 55 | 49* | 41* | 35* | 35* | 27* | 33* | 19* | 23* | NT |
| Background Neuropsychology | | | | | | | | | | | | | | |
| VOSP Screening | 20 | 15 | 19 | 19 | 20 | 20 | 20 | 18 | 18 | NT | 20 | NT | NT | 19 |
| VOSP Position Discrimination | 20 | 18 | 20 | 10* | 19 | 20 | 20 | 20 | 19 | NT | 20 | NT | NT | NT |
| VOSP Number Location | 10 | 7 | 10 | 10 | 6* | 10 | 10 | 6* | 10 | NT | 10 | NT | NT | NT |
| GNT | 30 | – | 3* | NT | 6* | 1 | 11* | 0 | 0 | 0 | 0 | 0 | 0 | 0 |
| MMSE | 30 | 26 | 28 | NT | 27 | 27 | 27 | 24 | 25 | 23 | 21 | 23 | NT | 15 |

Patients are arranged in order of their composite semantic scores derived from the three background tests of semantic processing (i.e., WPM, picture naming, CCTp). PM = word to picture matching; CCTp = picture version of the Camel and Cactus Test [7]; VOSP = Visual Object and Space Perception battery [26]; GNT = Graded Naming Test [49]; MMSE = Mini-mental State Exam [50]* denotes impaired performance. NT = not tested. TA = testing abandoned.

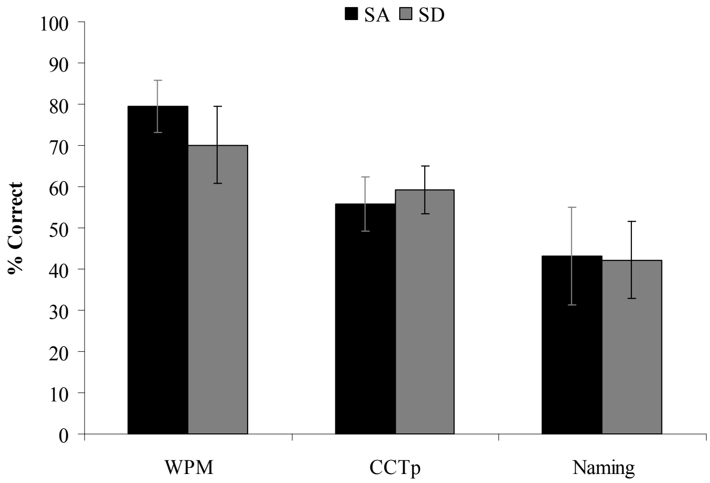

Fig. 2. Background semantic scores.

of executive/attentional control. Scores on the Brixton spatial anticipation test (number correct) and Raven's Coloured Matrices correlated with the composite semantic scores for this group (one-tailed: $r = 0.92, p < 0.001$; $r = 0.59$, $p < 0.05$, respectively), while the correlation between semantic severity and backwards Corsi approached significance ($r = 0.51$, $p = 0.08$ one-tailed).

## 4. Experimental task – Feature Reality Task

### 4.1. Method

Participants were presented with pairs of pictures and asked to indicate the 'more real-looking' of the two. The two pictures in each presentation differed along a single feature dimension (i.e., colour, environmental context, sound or motion). The real pictures were presented alongside either plausible or implausible distracter images. Plausible distracters were constructed by manipulating one of the target object's features such that it was typical of other exemplars from the targets semantic category. In contrast, implausible distracters possessed features which not were typical of the targets' neighbours. Thirty-three concrete concepts were used in this experiment, selected from a range of living and non-living categories. Each item appeared in at least two of the four experimental conditions (see Table 4). Specific details about each of the four conditions are provided below.

### 4.2. Condition 1: Colour

Twenty-nine concepts were presented as coloured line drawings. Each appropriately coloured line drawing was presented twice – once with a duplicate coloured in a typical way for that semantic category (e.g., *grey elephant* presented with a *brown elephant*) and once with an atypically coloured distracter (e.g., *pink elephant*).

### 4.3. Condition 2: Environmental Context

Twenty-nine pictures were superimposed on an environmental context. The real items were presented in their most common context (e.g., *horse* in a *field*). Distracter items were presented on either a plausible context (e.g. *horse* in a *desert*) or implausible context (e.g., *horse* in a *shopping centre*).

### 4.4. Condition 3: Sound

Twenty-six black and white line drawings were accompanied by sounds. Plausible distracter images were taken from the same domain as the target – and produced similar types of sound (e.g., the target *dog* presented alongside a picture of a *cat*). Pictures from the opposing semantic domain – which were associated with very different sounds – were used as implausible distracters (e.g., the target *dog* presented alongside a picture of an *alarm clock*).

### 4.5. Condition 4: Motion

Twenty concepts were presented as of pairs of animations, each showing the same item involved in two distinct kinds of motion. For example, a *cow* was depicted as chewing and swinging its tail from side to side in the target animation but as rearing up on its hind legs in the manner of a *horse*, in the distracter image. The plausibility manipulation was not incorporated into the motion test.

## 5. Results

### 5.1. Overall accuracy

Figure 3 shows the performance of the patients and controls on the four conditions of the feature reality task. A 3 (group) by 4 (condition) ANOVA revealed a main effect of group ($F$ (2,30) = 23.25, $p < 0.001$); post hoc comparisons showed the control group performed better than both patient groups, while SA patients performed better than SD patients (Bonferroni $p < 0.05$). There was no effect of condition.

Table 4
Familiarity ratings for items in the FRT

| Item | Mean Familiarity | Colour | Context | Sound | Motion |
|---|---|---|---|---|---|
| Apple | 4.9 | ■ | ■ | ■ | |
| Banana | 4.48 | ■ | ■ | ■ | ■ |
| Bee | 2.43 | ■ | | ■ | |
| Cat | 3.62 | ■ | ■ | ■ | ■ |
| Clock | 4.86 | | | ■ | ■ |
| Cow | 2.29 | ■ | ■ | ■ | ■ |
| Crab | 1.38 | ■ | ■ | | ■ |
| Dog | 3.52 | ■ | ■ | ■ | ■ |
| Duck | 2.48 | ■ | ■ | ■ | ■ |
| Egg | 4.47 | ■ | ■ | ■ | ■ |
| Elephant | 1.43 | ■ | ■ | ■ | |
| Fire Engine | 2.86 | ■ | ■ | ■ | |
| Frog | 2.14 | ■ | ■ | ■ | ■ |
| Guitar | 2.23 | ■ | ■ | ■ | |
| Hammer | 2.9 | | ■ | ■ | ■ |
| Helicopter | 2.57 | ■ | ■ | ■ | ■ |
| Horse | 2.86 | ■ | ■ | ■ | ■ |
| Kangaroo | 1.38 | ■ | ■ | ■ | ■ |
| Mouse | 1.9 | ■ | ■ | ■ | |
| Orange | 4 | ■ | ■ | | |
| Penguin | 1.38 | ■ | ■ | ■ | ■ |
| Piano | 3.33 | ■ | ■ | | ■ |
| Pig | 1.86 | ■ | ■ | ■ | |
| Pigeon | 3.76 | ■ | ■ | | |
| Saw | 2.33 | | ■ | ■ | ■ |
| Scissors | 4.48 | ■ | | ■ | ■ |
| Snowman | 2.1 | ■ | ■ | | |
| Suitcase | 3.76 | ■ | ■ | ■ | ■ |
| Tap | | | | ■ | |
| Tiger | 1.33 | ■ | ■ | ■ | |
| Tortoise | 1.38 | ■ | ■ | | ■ |
| Trumpet | 1.67 | ■ | ■ | ■ | |
| Windmill | 1.71 | ■ | ■ | | ■ |

### 5.2. Plausibility effects

The effect of distracter plausibility was investigated using a 3 (group) by 2 (distracter plausibility) ANOVA. Plausible distracters resulted in less accurate performance overall ($F$ (1,30) = 58.35, $p < 0.001$). This effect was most evident in the SD group, resulting in a group by plausibility interaction ($F$ (2,30) = 8.89, $p = 0.001$; see Fig. 4). Bonferroni comparisons confirmed that the plausibility effect was strongest in the SD group ($t$ (11) = 5.6, $p < 0.001$) but also present to a lesser extent in the SA ($t$ (8) = 4.1, $p < 0.01$) and control ($t$ (11) = 3.8, $p < 0.01$) groups.

Given the excellent performance of the SA patients, we also examined the effects of the plausibility manipulation on RT using a 2 (SA vs. control group) by 2 (distracter plausibility) ANOVA. Incorrect responses and outliers (outside 2 SDs) were not included in the analysis. Although RT did not differ significantly between groups ($F$ (1,19) < 1), there was a group by plausibility interaction ($F$ (1,19) = 8.1, $p = 0.01$, see Fig. 5). RT was not affected by distracter plausibility

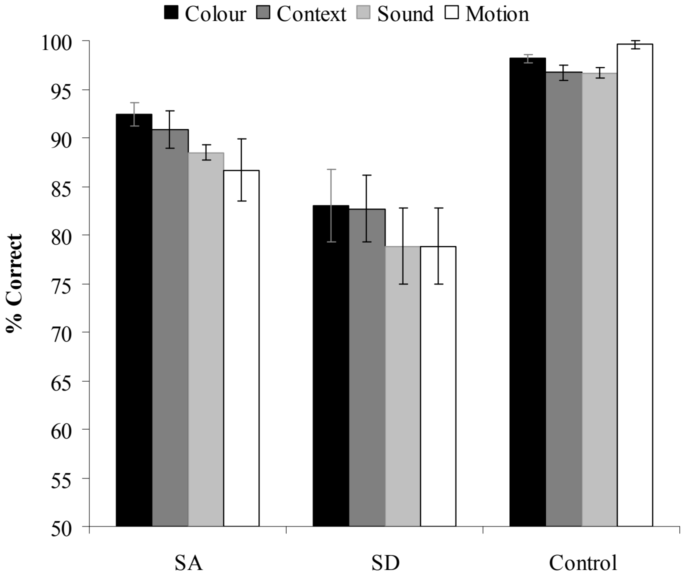

Fig. 3. Overall accuracy on the FRT.

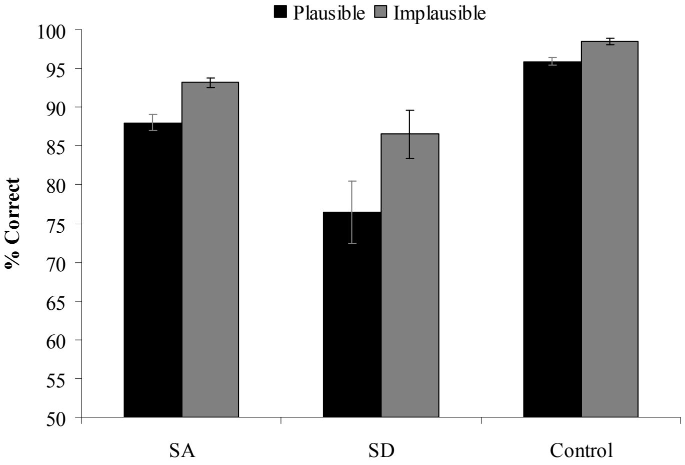

Fig. 4. Distracter plausibility and FRT accuracy.

in the SA group ($t < 1$), but a strong effect was present in the control group ($t$ (11) $= 4.1$, $p < 0.005$ Bonferroni corrected). Individually, none of the SA patients showed a significant difference between plausible and implausible trials ($t < 1$). In contrast, 7/12 controls were significant slower on plausible trials ($t = 3$–$1.6$, $p < 0.05$ one-tailed).

### *5.3. Correlations between FRT components and background tasks*

The SA group showed minimal correlations between the various sub-tasks within the FRT, in fact only one of six possible correlations was significant (i.e., motion and context: $r = 0.69$, $p < 0.05$). Furthermore, none of the FRT sub-tasks correlated with any of the three background tests of semantic knowledge (naming: $r < 0.24$, $p > 0.5$; WPM: $r < 0.34$, $p > 0.2$; CCTp: $r < 0.41$, $p > 0.1$) or the composite semantic severity score ($r < 0.16$, $p > 0.6$). A similar pattern of non-significant correlations were observed when overall performance on each FRT sub-task was compared with scores on the background executive measures ($r < 0.4$, $p > 0.1$). In contrast, the SD group showed strong correlations between 3/4 of the FRT sub-tasks – i.e.,

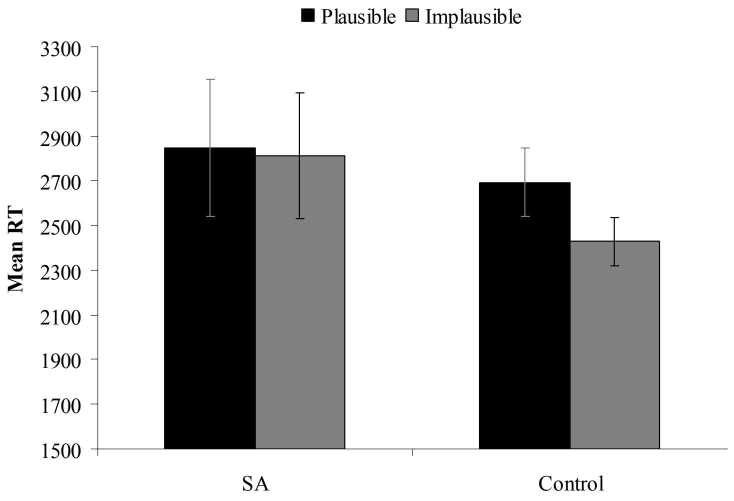

Fig. 5. FRT reaction times.

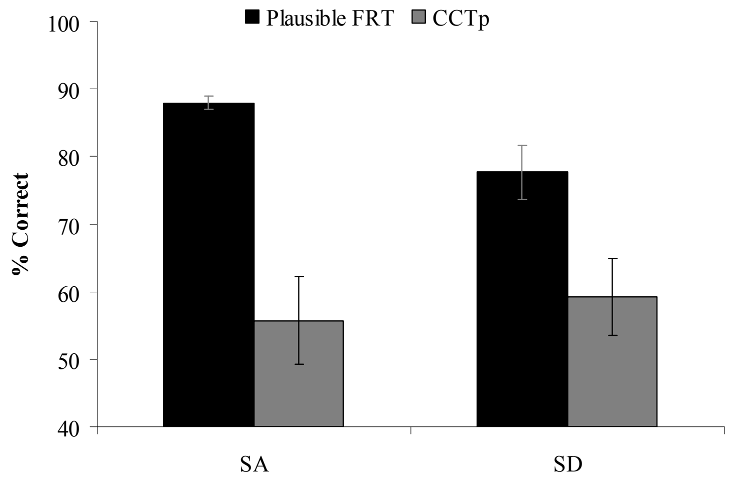

Fig. 6. Differential effects of regulatory demand on accuracy in SA and SD.

colour, context and sound all correlated with each other: $r > 0.73, p < 0.001$). These three conditions were also strongly correlated with composite semantic severity scores ($r > 0.69$, $p < 0.05$) and performance on each of the background semantic tasks ($r > 0.69, p < 0.05$). In contrast, the motion sub-task did not correlated with any of the other FRT sub-tasks or performance on the background semantic measures ($r = -0.29$–$0.13, p > 0.3$).

### 5.4. *The relationship between regulatory constraints and task performance*

To investigate whether the differential plausibility effects in SA and SD arose from the semantic control demands of the tasks, patient performance on plausible trials was directly compared with the two background receptive tasks (i.e., WPM and CCTp). The CCTp places higher demands on semantic elaboration and inhibitory control because it is necessary to work out which aspect of an object's meaning is relevant on any given trial. For example, we know many things about *camels* and *cacti* so selecting the correct response requires the ability to focus on the relevant desert relationship. In contrast, WPM involves more straightforward identify matching and therefore might place fewer demands on semantic control. The 2AFC format of the FRT task might also reduce demands on semantic control leading to better performance in the SA than the SD group. Therefore, a group by task interaction

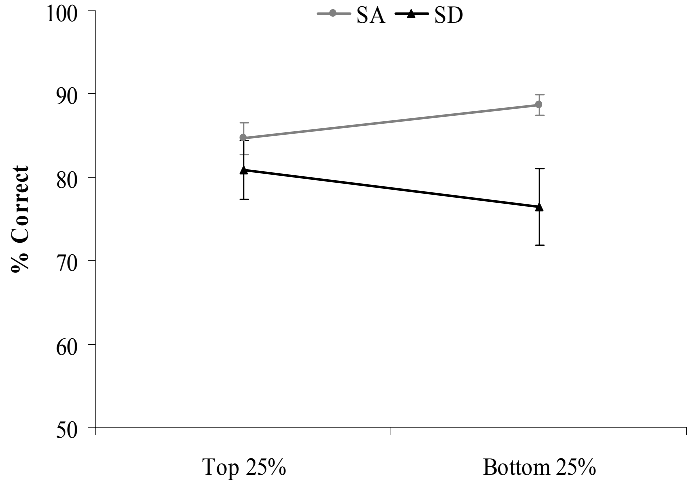

Fig. 7. Differential familiarity effects in SA and SD.

might be expected when the highly constrained FRT is compared with CCTp but not WPM. Both groups did better on the FRT task than the background measures ($F$ (1,18) = 54, $p < 0.001$). However, as predicted, a group by task interaction emerged when plausible FRT trials were compared with the CCTp ($F$ (1,18) = 4, $p = 0.06$). No interaction was present for the WPM comparison ($F$ (1,18) < 1). The results suggest that group differences in FRT performance are underpinned by the minimal regulatory requirements of this task, combined with relative preservation of the specific features of concepts in the SA vs. SD group.

### 5.5. Familiarity effects

Previous work indicated that SA patients are less influenced by familiarity/frequency than SD patients [36]. This issue was examined using a series of logistic regression analyses which aimed to predict individual item accuracy. A combined group analysis including, familiarity, patient group and a group by familiarity interaction term significantly predicted behaviour ($\chi^2$ (3) = 82.9, $p < 0.01$). Overall performance was influenced by both familiarity (Wald = 6.2, $p < 0.05$) and patient group (Wald = 35.96, $p < 0.001$), and a significant interaction emerged between these variables (Wald = 10.78, $p < 0.001$). Analysis on each group separately revealed that SA patients were influenced by familiarity (Wald = 5.16, $p < 0.05$), but counter intuitively, they were less likely to make an accurate judgement as item familiarity increased (Exp(B) 0.84, C.I.$_{95}$, 0.74–0.97). In contrast, SD patients showed the opposite pattern; the odds of making a correct judgement increased with item familiarity (Exp(B) 1.13, C.I.$_{95}$, 1.07–1.24, Wald = 6.22, $p = 0.05$). In each of the above models, the predictive power and directionality of the familiarity effect was unaffected by the inclusion of distracter plausibility and patient identity as independent predictors (i.e., Wald values for familiarity remained within a similar range: 9.42–3.26, $p < 0.05$). A separate ANOVA analysis comparing performance on the most and least familiar items (i.e., top and bottom 25% of items) also yielded a group by familiarity interaction ($F$ (1,19) = 5.2, $p < 0.03$, see Fig. 7). SA patients were less accurate on the high frequency items ($t$ (8) = 1.9, $p = 0.04$ one tailed), while the opposite pattern approached significance in the SD patients ($t$ (11) = 1.7, $p = 0.06$ one tailed).

## 6. Discussion

This study investigated the nonverbal semantic performance of two groups of patients with multimodal semantic impairment, semantic aphasia (SA) and semantic dementia (SD), on a reality decision judgement task. The patients were required to select the correct colour, context, sound or motion for objects in a two alternative forced choice (2AFC) test. In addition, three of these tasks manipulated the plausibility of distracter items. Plausible distracters were typical of the object's semantic category (e.g., a *red tomato* vs. a *green tomato*, which is the typical colour for plants). Therefore, this manipulation examined the ability to base judgements on the specific features of objects while avoiding incorrect shared features. By directly comparing the SA and SD groups on these tasks, this study of nonverbal semantic processing casts light on the nature of semantic deficits that follow left frontal and/or temporoparietal infarcts in SA and anterior temporal lobe atrophy in SD. It has been suggested that the semantic impairment in these groups has a different underlying cause: deregulated semantic control in SA and degraded conceptual representations in SD [1]. As a consequence, SA patients might be less affected by the plausibility manipulation than the SD group; their intact semantic representations and the relatively constrained 2AFC test format should allow relatively accurate performance. SD patients, in contrast, are highly sensitive to the plausibility manipulation due to their loss of specific fine-grained knowledge [14,15]. The results of this study upheld these predictions and highlighted significant differences between the semantic profiles of SA and SD. The main points are summarised below:

1. SA patients performed better than SD on reality decision judgements even though both groups performed similarly on background tests of semantic knowledge.
2. The SA patients were relatively insensitive to the plausibility manipulation in both accuracy and RT, in sharp contrast to the SD patients.
3. The type of semantic processing required in a task strongly influenced SA patients' performance, whereas SD patients were consistent across all measures.
4. SD patients showed a beneficial affect of familiarity. In contrast, SA patients were *less* likely to produce a correct response as item familiarity increased.

This differential performance can be explained by considering the aetiology of each group's semantic impairment coupled with the regulatory constraints imposed by the semantic tasks.

### 6.1. Reality decision judgements in SD

SD patients present with a highly specific and progressive impairment of semantic memory which follows atrophy and hypometobolism focussed on the bilateral ATL [6,37]. They fail a broad range of verbal and nonverbal semantic measures regardless of the nature of the stimuli [i.e., words, pictures, sounds, and smells; 7, 9]. SD patients show a profile of semantic performance which is consistent with degraded representations being the causal underlying deficit – they show strong item consistency across different semantic tasks and minimal effects of cueing [7,13]. One key additional feature of their profile helps to explain the current findings; In SD the loss of fine-grained semantic knowledge (e.g., the knowledge that *strawberries* are *red*) is more vulnerable than more general superordinate information (e.g., *strawberries* are *plants*). Patients show greater difficulties on tasks that require access to specific semantic knowledge, such as sorting pictures into specific vs. general categories [15]. Moreover, when drawing items from memory, SD patients not only omit idiosyncratic features (e.g., drawing a rhino without its horn) but also make intrusion errors, including features which are typical of other category members [e.g., drawing a duck with four legs; 38]. With the progressive loss of specific features (e.g., the *rhino's* horn), the semantic system is left representing category structure in terms of the most frequently encountered similarities shared across category members [15,17]. This is why the SD group are highly sensitive to the plausibility manipulation when making reality judgements in this study. When the target (e.g., *red strawberry*) is presented alongside a plausible distracter (e.g., *green strawberry*), SD patients are likely to select the stimulus which has the largest overlap with the remaining similarity structure coded in the partially degraded semantic network. Implausible distracters (e.g., *blue strawberry*) share less similarity with category members – because their features are generally not typical for other semantic neighbours – therefore, they can be more easily rejected. Consequently, our results support the hypothesis that the bilateral ATL is a critical substrate for semantic representations [3,39,40] and degeneration of this bilateral network leads to the large effects of plausible distracters seen on the FRT task. In SA, however, the ATL is spared and patients do not show a profile consistent with degraded semantic knowledge. So what can account for the multimodal comprehension impairment in this group?

### 6.2. Reality decision judgements in SA

SA patients present with a number of characteristics which suggest their impaired performance stems from an inability to regulate conceptual knowledge as opposed to degraded semantic representations per se. SA patients are highly sensitive to the executive demands of semantic tasks – indeed, on a range of measures, their performance has been shown to reflect the ease with which target items can be selected whilst competing information is ignored [1,4,5,11] Similarly, item consistency – the ability to access the same concept on different occasions – is low when the nature of the task changes (e.g., naming vs. associative decisions). Finally, semantic impairment in SA correlates with domain-general executive/control problems – as measured by standardised tests of reasoning and cognitive flexibility [i.e., Ravens Coloured Matrices and the Wisconsin Card Sorting Test; 1]. In this study, the SA patients performed comparatively well on the experimental task because it placed minimal demands on self-generated regulation of semantic activation. The 2AFC format provided strong constraints on the target response and helped to minimise competition from accompanying choices. The insensitivity of the SA patients to the plausibility of distracters suggests that the specific features of items were not severely degraded, in contrast to SD. Previous investigations of cueing effects support this interpretation by showing that SA patients are much better at retrieving semantic information when their response space is constrained, even when the task requires access to specific semantic knowledge [5,13]. Our findings are, therefore, consistent with the interpretation that SA patients' comprehension problems arise from difficulties regulating and shaping activation. The extent to which this impairment will affect overall performance is mediated by the nature of the task and the amount of constraint it places on intrinsic semantic control.

The deficient semantic control hypothesis is consistent with the nature of the lesions in the SA group which affect left PFC and TPJ regions [1,19]. These areas show coupled activation on a range of executive and attentional tasks in functional neuroimaging studies [41,42]. Similarly, they are both activated when semantic tasks place a high demand on regulatory control – studies looking at the resolution of conflict when dealing with ambiguous material frequently show activation in these two areas [22,23,43], while additional studies have suggested an important role of inferior frontal and/or temporoparietal areas in the selection/retrieval and allocation of attention to semantic representations [21,44,45].

### *6.3. Differential effects of familiarity in SA & SD*

We found that SA patients were less likely to make correct judgements as item familiarity increased, while SD patients showed better performance with increasing item familiarity. A number of previous investigations have shown that SA patients are insensitive to familiarity/frequency. In their original case-series comparison, Jefferies & Lambon Ralph [1] found the SA group showed no familiarity effects on any of the semantic tests, a finding replicated in subsequent work on refractory effects [12]. Conversely, strong effects of familiarity/frequency are consistent characteristics of SD [1, 46]. The benefits of familiarity/frequency in SD can be explained with reference to the underlying principles of PDP models. Items which are encountered frequently present the model with greater opportunity to learn and distinguish between similar exemplars [17,47]. In contrast, familiarity/frequency may have a negative effect in SA by increasing regulatory demand. Recent studies have shown that highly familiar/frequent items are generally encountered in a larger variety of environmental contexts, therefore regulatory control is required to focus selectively on the relevant aspects of knowledge that are appropriate for a given task [36].

## 7. Conclusions

The nature of the semantic impairment in SA and SD interacted with the regulatory demands of the FRT to produce qualitatively different semantic performance in the two groups of patients. The SA patients, supported by their intact semantic knowledge and a task structure which reduced competition at an individual trial level, performed well overall and showed minimal effects of the feature plausibility manipulation. However, their underlying regulatory impairment emerged in their reversed sensitivity to familiarity. SD patients in contrast, exhibited a profile which was best explained in terms of degraded semantic knowledge; they were highly sensitive to the feature plausibility manipulation and performed better on high familiarity items. This provides further support for the proposal that semantic cognition is underpinned by two principle components: semantic representations and regulatory control processes which regulate and shape activation within the semantic system.

## Acknowledgments

We are indebted to the patients and their carers for their generous assistance with this study. We would like to thank Rachel Byrne, Linda Collier and Claire Slinger for referring some of the patients to us. The work was supported by a programme grant from the MRC (G0501632) and an MRC studentship to KAN.
